# T-cell help in the germinal center: homing in on the role of IL-21

**DOI:** 10.1093/intimm/dxad056

**Published:** 2024-01-02

**Authors:** Lina Petersone, Lucy S K Walker

**Affiliations:** University College London Division of Infection and Immunity, Institute of Immunity and Transplantation, Pears Building, Royal Free Campus, London NW3 2PP, UK; University College London Division of Infection and Immunity, Institute of Immunity and Transplantation, Pears Building, Royal Free Campus, London NW3 2PP, UK

**Keywords:** autoimmunity, B cells, follicular helper T cells (Tfh), humoral immunity

## Abstract

Interleukin 21 (IL-21) is a pleiotropic cytokine that is overproduced in multiple autoimmune settings. Provision of IL-21 from follicular helper T cells is an important component of T-cell help within germinal centers (GC), and the last few years have seen a resurgence of interest in IL-21 biology in the context of the GC environment. While it has been more than a decade since T cell-derived IL-21 was found to upregulate B-cell expression of the GC master transcription factor B-cell lymphoma 6 (Bcl-6) and to promote GC expansion, several recent studies have collectively delivered significant new insights into how this cytokine shapes GC B-cell selection, proliferation, and fate choice. It is now clear that IL-21 plays an important role in GC zonal polarization by contributing to light zone GC B-cell positive selection for dark zone entry as well as by promoting cyclin D3-dependent dark zone inertial cycling. While it has been established that IL-21 can contribute to the modulation of GC output by aiding the generation of antibody-secreting cells (ASC), recent studies have now revealed how IL-21 signal strength shapes the fate choice between GC cycle re-entry and ASC differentiation *in vivo*. Both provision of IL-21 and sensitivity to this cytokine are finely tuned within the GC environment, and dysregulation of this pathway in autoimmune settings could alter the threshold for germinal center B-cell selection and differentiation, potentially promoting autoreactive B-cell responses.

## Teaser

Novel roles for IL-21 in regulating germinal centers

## Introduction

Interleukin 21 (IL-21) was first identified in 2000 as a soluble 162 amino acid-long polypeptide derived from activated human T cells that promoted *in vitro* proliferation of a cell line engineered to express the newly discovered IL-21 receptor (IL-21R) (1, 2). Subsequent studies have since established IL-21 to be a highly potent and pleiotropic immunomodulator with diverse roles in regulating both the innate and the adaptive arms of the immune system (3–5).

The broad effects of IL-21 signaling *in vivo* are facilitated by the widespread expression of its receptor on cells of both myeloid and lymphoid lineages including CD4+ and CD8+ T cells, B cells, natural killer cells, natural killer T (NKT) cells, and dendritic cells, as well as non-immune cells, including fibroblasts, keratinocytes, and gastric epithelial cells (1, 6–12).

The receptor for IL-21 comprises an IL-21R subunit which is structurally closely related to the IL-2R β and IL-4R α chains and forms a heterodimer with the common cytokine receptor γ chain (1, 2). In line with the molecular composition of its receptor, IL-21 belongs to the type I cytokine family (1) and signals via Janus kinase (JAK) 1 and JAK3 proteins, which phosphorylate signal transducer and activator of transcription (STAT) 1, STAT3, and to a lesser extent STAT4 and STAT5 (13–15). Furthermore, IL-21 can also induce activation of phosphoinositide 3-kinase (PI3K) and mitogen-activated protein kinase (MAPK) pathways (16, 17). This diverse IL-21 signaling is highly context-dependent and has been shown to both activate and inhibit immune responses [reviewed in (18)].

While IL-21 can also be produced by NKT cells (9) and CD8+ T lymphocytes (19), several landmark studies have pinpointed CD4+ T helper 17 (Th17) and follicular helper T (Tfh) cells as the main sources of IL-21 *in vivo* (8, 20, 21). Importantly, IL-21 has now been recognized as the archetypal cytokine of the Tfh cell subset (8, 20, 22), and the last 20 years of research have firmly established IL-21 as a key regulator of the adaptive immune response. In this review, we highlight the progress made in this field, from the very first observations linking IL-21 with *in vivo* immunoglobulin (Ig) production to the most recent studies providing more granular insights into the molecular mechanisms underpinning IL-21-dependent germinal center (GC) regulation.

## Identification of IL-21 as a key regulator of humoral immunity

IL-21 was first implicated in the modulation of humoral immunity by Ozaki and colleagues who demonstrated that IL-21 greatly promoted differentiation of plasma cells and production of class-switched antibodies following a T-dependent antigen challenge in mice (Table 1) (23, 28). The decreased frequencies of class-switched memory B cells and lowered serum IgG levels in people with IL-21 deficiency (29) or IL-21R loss-of-function mutations (30–32) confirm that IL-21 also represents an essential regulator of humoral immunity in humans.

**Table 1.** Early evidence of IL-21 contributing to the regulation of humoral immunity.

Evidence	References
IL-21R−/− mice display normal proportions of monocyte, lymphocyte, and granulocyte populations	(23, 24)
IL-21R−/− and IL-21−/− mice exhibit altered immunoglobulin production (reduced IgG1, IgG2b, IgG3, IgA, and increased IgE levels)	(23–27)
IL-21 promotes BLIMP1 expression and plasma cell differentiation in murine B cells	(26, 28)
IL-21 promotes Bcl-6 expression in murine B cells	(25, 28)
IL-21R−/− and IL-21−/− mice form smaller GC that dissolve faster than wild-type GC	(25–27)
IL-21 promotes B-cell affinity maturation	(25, 26)

IL-21 signaling was further linked to GC regulation by Chtanova *et al*. (8) who reported that Tfh cells from human tonsils were characterized by high IL-21 gene expression and Vinuesa and colleagues (20) confirmed that high IL-21 production was also a feature of the murine Tfh cell compartment. Subsequent work showed that IL-6 and IL-12 appear to be important cytokines for promoting T cell IL-21 production (33, 34), and further *in vitro* investigations verified that IL-21 secreted by Tfh cells could indeed support the capacity of B cells to generate antibody (35, 36).

*In vivo* studies in IL-21−/− and IL-21R−/− mice demonstrated that B cell-intrinsic IL-21 signaling markedly increased the expression of the GC B-cell master transcriptional regulator B-cell lymphoma 6 (Bcl-6) (Table 1) (25, 26) which is indispensable for GC formation (37). These studies also revealed that even though GC could form in the absence of IL-21 signaling, GC structures in mice lacking IL-21 or IL-21R expression were smaller in size, showed decreased B-cell proliferation, and dissolved sooner than those in their wild-type counterparts (25–27, 38).

The effects of IL-21 modulation were found to extend beyond the boundaries of the GC and into the memory compartments, highlighting IL-21 as a key player in establishing robust and lasting humoral immune responses. While analyses of memory B-cell populations found that, during primary immune challenges, memory B cells could form even in the absence of intact IL-21 signaling (26), IL-21R deficiency impaired memory B-cell accumulation during a secondary challenge (39). Memory B cells are characterized by a high degree of functional and phenotypic heterogeneity with different populations following distinct fates during recall responses (40). Interestingly, IL-21R expression was shown to be higher in a memory B-cell subset associated with secondary GC formation but not amongst memory B cells linked to recall plasma cell responses (41) which may indicate a more nuanced role for IL-21 in shaping memory B-cell differentiation and function.

**Figure 1.**
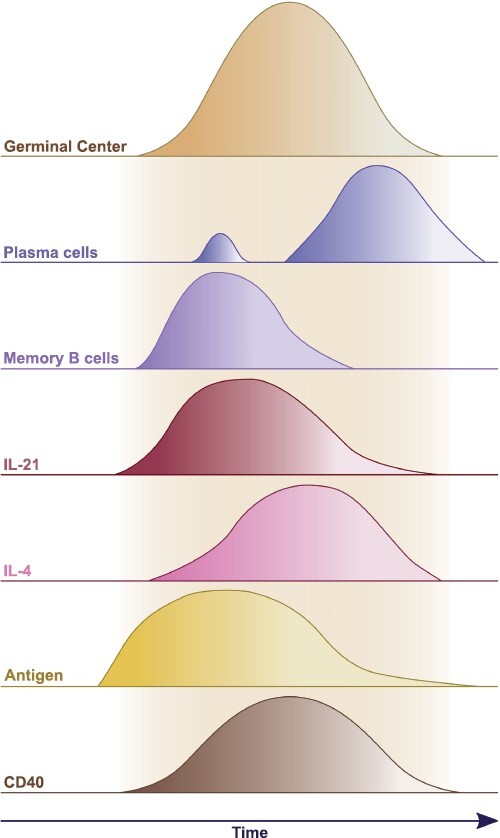
Kinetics of GC B-cell differentiation and GC modulatory signals. A cartoon depicting the timelines of GC output kinetics and temporal changes in signals regulating GC processes. While some plasma cells differentiate shortly after GC formation (38), most of the long-lived plasma cells are formed late during the GC response (45). Memory B cells differentiate early into the GC response (45). IL-21 production is initiated during the earliest stages of GC formation (46, 47). IL-4 production is preserved in late-stage GC (46, 47). Antigen is abundantly available at the start of the GC response and is preserved late into the GC response by central light zone follicular dendritic cells (48). CD40 signaling is essential for GC maintenance throughout the GC response (49).

IL-21 has also been shown to promote the expression of the plasma cell master transcription factor B-lymphocyte-induced maturation protein 1 (BLIMP1), and IL-21 has been identified as an essential differentiation factor for GC-derived plasma cells (25, 26, 28, 38, 42, 43). Differentiation of plasma cells is known to require extensive B-cell proliferation (44). Furthermore, while memory B-cell formation is largely seen during the earliest stages of a GC response, differentiation of long-lived plasma cells largely occurs later in the GC process (Fig. 1) (45). Therefore, the defects in plasma cell formation reported in the absence of IL-21 signaling may in part be linked to the aforementioned roles of IL-21 in promoting B-cell proliferation and GC maintenance (25, 26).

In addition to regulating the scale of GC responses and the composition of the GC-derived plasma and memory B-cell pools, IL-21 signaling was also found to shape the quality of the GC output. Zotos and colleagues (26) observed significantly reduced frequencies of somatic hypermutation (SHM) and affinity-enhancing amino acid exchange events in immunoglobulin genes of immunogen-specific memory B cells from animals with defective IL-21 signaling. Similarly, the affinity of the GC-derived plasma cell compartment was also found to be substantially lower in IL-21- and IL-21R-deficient mice (25, 26). Thus, the IL-21 pathway plays an important role in affinity maturation.

## IL-21 promotes GC expansion and shapes GC B-cell polarization

It was initially suggested that IL-21 was required for Tfh cell differentiation (50, 51), however, subsequent work established that Tfh cells could still form in animals with defective IL-21 signaling (25, 26, 52). Nevertheless, IL-21 has been shown to support Tfh cell expansion and maintenance (25, 53, 54) and it has a marked impact on the ratio of Tfh to follicular regulatory T cells (Tfr) in transient as well as autoimmune GC (55, 56).

A more profound impact of IL-21 signaling is seen in B cells. Recent work by Dvorscek (57) and colleagues has reinforced the importance of IL-21 as a key modulator of early B-cell proliferation during T cell-dependent immune responses. Elegant bromodeoxyuridine (BrdU) pulse experiments demonstrated that IL-21 increased both the rate of B-cell cycle re-entry as well as the speed at which they underwent division. A study by Zotos *et al*. (58) confirmed that IL-21 also acted as a key driver of GC B-cell proliferation in established GC. Further dissection of the molecular mechanisms underlying IL-21-dependent acceleration of B-cell proliferation revealed that IL-21 possessed a pronounced ability to enhance B-cell receptor (BCR)- and CD40-driven phosphorylation of AKT and ribosomal protein S6 (57, 58). AKT and S6 are both part of the mammalian target of rapamycin complex 1 (mTORC1) pathway and mTORC1 activation is central to the metabolic reprogramming that needs to be initiated in GC B cells to support their rapid and extensive clonal expansion (59).

The ability of IL-21 to synergize with CD40 in inducing S6 phosphorylation in GC B cells *in vitro* was further confirmed in recent studies by the Shlomchik and Yu groups (60, 61). Notably, Dvorscek *et al*. (57) demonstrated that the addition of IL-21 allowed B-cell division in the presence of low levels of CD40 stimulation that failed to induce noteworthy B-cell proliferation alone, suggesting that IL-21 signaling may expand the early GC B-cell repertoire by lowering the threshold of T cell engagement required for cell cycle progression.

**Figure 2.**
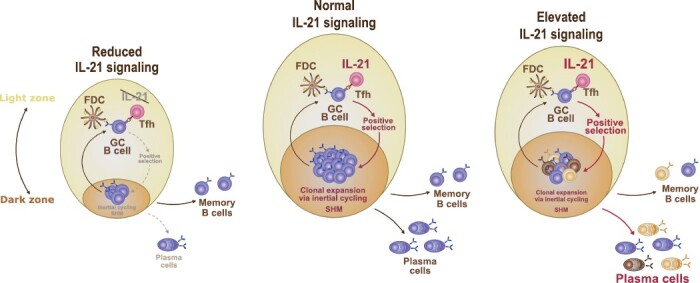
IL-21 regulates GC polarization and differentiation. Models depicting effects of changes in IL-21 signaling on GC dark zone/light zone ratios and GC output. GC with reduced or no IL-21 signaling (left) have reduced dark zone compartments and yield fewer plasma cells than GC with normal IL-21 levels (middle) (25, 26, 38, 54, 58, 64). Elevated IL-21 levels (right) lower the B-cell positive selection threshold and allow more B-cell clones to enter the GC dark zone (54, 60, 61). Exacerbated IL-21 production favors GC B-cell differentiation over GC maintenance (61). FDC, follicular dendritic cell; GC, germinal center; SHM, somatic hypermutation; Tfh, follicular helper T cell.

A major site for B-cell proliferation within the GC environment is the dark zone, where centroblasts undergo SHM before returning to the light zone for competitive antigen capture and T cell-dependent selection. While GC are typically dominated by dark zone cells in both mice and humans (62, 63) several studies have now noted marked skewing towards a light zone phenotype in GC B cells from IL-21- and IL-21R-deficient mice (Fig. 2) (46, 54, 55, 57, 58, 64). Defects in IL-21 signaling have been found to result in impaired dark zone compartments in immunization-induced GC (54, 57, 58) as well as in GC triggered by autoimmunity (54) and viral infection (64). Furthermore, IL-21 has been shown to modulate GC dark zone formation in both mice with wild-type polyclonal (54, 58) and restricted transgenic T cell and B-cell receptor repertoires (46, 57). The identification of a role for IL-21 in GC B-cell proliferation, and GC dark zone formation helps reconcile the defective GC expansion, maintenance, and reduced affinity maturation that have long been observed in animals with impaired IL-21 signaling (Table 1).

## IL-21 regulates GC B-cell positive selection

The GC dark zone is seeded by light zone GC B cells that have received Tfh cell-derived affinity-based positive selection signals (62). Positively selected light zone GC B cells upregulate the expression of transcription factor c-Myc (65, 66) and the extent of c-Myc upregulation was found to correlate with the extent of dark zone GC B-cell expansion (67). Further investigations into the phenotype of recently selected light zone GC B cells demonstrated that in addition to c-Myc, recently selected light zone GC B cells also upregulate the expression of transcription factors interferon regulatory factor 4 (IRF4) and basic leucine zipper ATF-like transcription factor (BATF) and show marked activation of the mTORC1 pathway (59, 65, 66, 68). More recently, the induction of BATF and mTORC1 has been recognized as a marker of T cell-instructed metabolic “refueling” required for sustained dark zone GC B-cell expansion (69). Several years ago, Luo *et al*. (70) demonstrated that BCR and CD40 signals could synergize to upregulate c-Myc expression and promote positive selection in GC B cells, however, if and how IL-21 contributed to light zone GC B-cell positive selection remained unclear.

**Figure 3.**
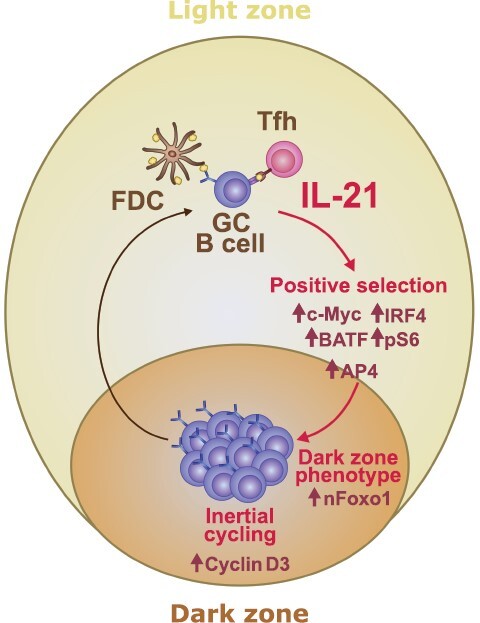
Mechanisms of IL-21-dependent GC regulation. A model depicting GC pathways that are modulated by IL-21 signaling. IL-21 promotes light zone GC B-cell positive selection by upregulating transcription factor c-Myc and IRF4 and supporting T cell-instructed B-cell metabolic “refueling” via stimulation of BATF and mTORC1 pathways (pS6) (54, 57, 58, 60, 61). IL-21 sustains c-Myc-induced B-cell proliferation by preserving the expression of transcription factor AP4 that functions downstream of c-Myc (71). IL-21 promotes dark zone centroblast differentiation by promoting nuclear localization of transcription factor Foxo1 (54). IL-21 tunes the inertial cycling of dark zone GC B cells by regulating the expression of cell cycle regulator cyclin D3 (54). AP4, activating enhancer-binding protein 4; BATF, basic leucine zipper ATF-like transcription factor; FDC, follicular dendritic cell; GC, germinal center; IRF4, interferon regulatory factor 4; nFoxo1, nuclear Foxo1; pS6, phosphorylated ribosomal protein S6; Tfh, follicular helper T-cell.

Recent highly complementary studies by the Shlomchik and Yu groups demonstrated that IL-21 could synergize with both CD40 and BCR signaling to enhance GC B-cell c-Myc expression *in vitro* (60, 61). Additionally, further analysis by Chen *et al*. (61) demonstrated that IL-21 could also promote the expression of IRF4 and BATF in B cells. Thus, IL-21 appeared to be capable of instilling the hallmarks of GC light zone B-cell positive selection. To investigate the effects of IL-21 signaling on GC B-cell positive selection *in vivo*, in our own recent study we devised a flow cytometry staining panel to track selected light zone GC B cells on the basis of their c-Myc, IRF4, and BATF expression (54). We were able to identify a distinct light zone GC B-cell population that co-expressed c-Myc, IRF4, and BATF, was dependent on CD40 signaling, and expressed elevated levels of phosphorylated ribosomal protein S6 (pS6) consistent with T cell-dependent selection. Following immunization, this c-Myc+IRF4+BATF+ light zone GC B-cell population changed in size in line with the overall GC kinetics in wild-type mice but was virtually absent in IL-21R−/− animals. Analysis of mixed bone marrow chimeric mice confirmed that this effect was B cell-intrinsic. Together with the aforementioned findings (54, 60, 61), these data provide strong evidence of a key role for IL-21 in light zone GC B-cell positive selection (Fig. 3).

## IL-21 drives GC B-cell inertial cycling

Following positive selection in the GC light zone, GC B cells transition into the GC dark zone compartment where they undergo extensive clonal proliferation (72). The number of divisions completed by a single dark zone GC B clone has been shown to be directly proportional to the amount of c-Myc induced during light zone GC B-cell positive selection (67). However, the expression of c-Myc protein is transient and largely lost upon GC B-cell migration into the dark zone compartment (62, 65, 66, 73) indicating that factors downstream of c-Myc rather than c-Myc itself are responsible for sustaining dark zone GC B-cell proliferation *in situ*. Work by Chou *et al*. (71) identified the transcription factor activating enhancer-binding protein 4 (AP4) as one such modulator acting downstream of c-Myc to promote cell cycle re-entry in dark zone GC B cells. This study demonstrated that exposure to IL-21 could enhance CD40-mediated AP4 expression *in vitro*. Furthermore, IL-21 but not IL-4 or IFN-γ was found to be able to preserve AP4 protein expression even after CD40 ligation was stopped. Consistent with these observations, IL-21R-deficient light zone GC B cells failed to upregulate AP4 *in vivo* while their dark zone counterparts displayed a markedly reduced proliferative capacity suggesting that IL-21 could regulate GC dark zone expansion via modulation of AP4 (Fig. 3).

As the proliferation of dark zone GC B cells occurs in the absence of further antigen-derived signals or T cell help it has been termed inertial cycling (74). While c-Myc expression “charges” light zone GC B cells for expansion in the dark zone compartment in a dose-dependent manner, the inertial cell cycle re-entry in dark zone GC B cells requires upregulation of cell cycle regulator cyclin D3 (74, 75). Elegant experiments revealed that cyclin D3 deficiency in GC B cells leads to a profound loss of the GC dark zone compartment and this defect cannot be overcome by enhanced c-Myc expression (74, 75). Intriguingly, the dark zone GC B-cell defect in cyclin D3 deficient cells bears a striking resemblance to that seen in animals with defective IL-21 signaling (54, 57, 58). When we investigated whether IL-21 could promote GC dark zone expansion via regulation of cyclin D3 levels, we were able to demonstrate that IL-21 could promote B-cell cyclin D3 expression *in vitro*, and more importantly, that IL-21R-deficient dark zone GC B cells failed to upregulate cyclin D3 expression *in vivo* (54).

ChIPseq analysis by Chou *et al*. (71) has demonstrated that cyclin D3 in GC B cells is a direct target of AP4, therefore, the recently described IL-21-dependent cyclin D3 upregulation may be in part due to IL-21-enhanced c-Myc and AP4 expression. In addition to AP4, the transcription factor forkhead box protein O1 (Foxo1) has also been shown to regulate cyclin D3 gene expression in B cells (75, 76). Foxo1 expression in GC B cells instructs the dark zone GC B-cell genetic program, including upregulation of the dark zone homing chemokine receptor CXCR4, and Foxo1 ablation in B cells results in a profound loss of the GC dark zone compartment (76, 77). BCR crosslinking has been shown to displace nuclear Foxo1 into the cytoplasm thereby ceasing dark zone gene transcription upon GC B-cell light zone re-entry (70). However, nuclear Foxo1 can again be readily detected amongst c-Myc+ light zone GC B cells (77) indicating a link between light zone GC B-cell positive selection and initiation of dark zone gene expression. Intrigued by the similarities between the GC dark zone defects associated with Foxo1, cyclin D3, and IL-21/IL-21R deficiencies (46, 54, 55, 57, 58, 64, 74–77), we examined whether IL-21 could support GC dark zone formation via modulation of Foxo1. Our findings revealed that IL-21R deficiency resulted in a marked reduction in GC B-cell Foxo1 levels and that IL-21 had the capacity to support B-cell Foxo1 nuclear localization (54). Furthermore, we showed that inhibition of Foxo1 transcriptional activity could dampen the extent of IL-21-driven cyclin D3 upregulation. Collectively these findings indicate a second pathway through which IL-21 may modulate GC dark zone formation and expansion.

Together, these studies position IL-21 as a key molecular link between light zone GC B-cell positive selection and their acquisition of a GC dark zone transcriptional program with the concomitant inertial cycling (Fig. 3) (54, 60, 61, 71).

## IL-21 signal strength modulates GC B-cell differentiation

The role of IL-21 in promoting the generation of antibody-secreting cells (ASC) in response to T-dependent antigens has long been recognized (26, 28, 38, 42, 43). However, whether this effect was achieved by IL-21 promoting GC maintenance and increasing B-cell proliferative capacity, both of which have been associated with plasma cell development (57, 70, 78), or whether IL-21 could directly instruct ASC differentiation program *in vivo* remained incompletely understood.

An elegant study by Luo *et al*. (60) recently addressed the effects of IL-21 signaling on GC B-cell differentiation and demonstrated that when combined with CD40 stimulation, IL-21 could readily direct GC B-cell differentiation into plasma cells *in vitro* whereas commitment to plasma cell fate was not observed when CD40 stimulation was delivered on its own or in combination with BCR signaling. Notably, this effect was not simply attributable to increased GC B-cell proliferation in the presence of IL-21 because the authors could observe higher plasma cell differentiation in cells that received CD40 and IL-21 stimulation even when compared to CD40 or CD40/BCR stimulated cells that had undergone the same number of divisions. Subsequent *in vivo* experiments further confirmed that concerted IL-21 and CD40 stimulation was central to the initiation of plasma cell differentiation amongst GC B cells. A complementary study by Chen and colleagues (61) highlighted IL-21 signal strength as a key modulator of ASC differentiation by demonstrating that attenuated IL-21 signaling leads to impaired plasma cell formation while enhanced IL-21 stimulation skews GC B cells away from GC recycling and towards ASC differentiation (Fig. 2). Furthermore, GC B cells from animals treated with exogenous IL-21 were found to display markedly increased clonal diversity and reduced affinity maturation that was mirrored by a significantly lower affinity of their serum antibody pool. Thus, IL-21 signaling emerges as a central modulator of GC B-cell selection and differentiation thresholds.

Of note, Luo *et al*. (60) found that IL-21 together with CD40 stimulation was also a potent inducer of memory B-cell differentiation and this effect was further enhanced by the addition of BCR stimulation. This highlights the complex interplay between signaling pathways regulating GC B-cell fate decisions and emphasizes the need for further investigations into their relative involvement and temporal changes throughout the GC response.

## The interplay between IL-21 and IL-4 in GC regulation

The growing body of evidence pinpointing IL-21 as a key regulator of GC processes has raised questions about whether other Tfh cell-derived cytokines may play similar roles in GC modulation. The interplay between IL-21 and IL-4 has been of particular interest since both cytokines are readily produced by Tfh cells within the GC (38, 46, 47) and have been found to synergize in supporting GC-derived antibody production (23, 79).

However, despite some level of cooperation between IL-21 and IL-4 in supporting humoral immune responses, studies on the roles and regulation of both pathways have demonstrated profound differences between these two Tfh cell-derived cytokines. IL-21R signaling within GC B cells is rewired towards a robust yet transient p-STAT3 induction (60), yielding a considerable degree of similarity between IL-21R- and STAT3-deficient GC B-cell responses (60, 80). Conversely, IL-4 signal activates the STAT6 pathway (81).

IL-21-producing Tfh cells have been shown to emerge early on during T-dependent immune responses consistent with the role of IL-21 in driving early GC B-cell proliferation, whereas IL-4-producing Tfh cells appear to accumulate once the GC is established (Fig. 1) (46, 47, 57). Molecular modulators of Tfh cell differentiation and effector function include a complex network of cytokines and co-stimulatory molecules (82) and their contributions to the temporal regulation of Tfh cell IL-21 and IL-4 production remain active areas of research. Interestingly, IL-21 and IL-4 signaling appear to display opposing effects on GC polarization, with IL-21 playing an integral role in the formation of the GC dark zone compartment (46, 54, 55, 57, 58, 64) and IL-4 signals augmenting the size of the GC light zone compartment (46, 83). Notably, the transcriptional profiles and *in situ* localization of IL-21- and IL-4-producing Tfh cells are markedly different, providing a further indication of the non-redundant roles for IL-21 and IL-4 in GC regulation (47). Intriguingly, recent work by Chen *et al*. (61) revealed that the expression of the enzyme Ndst1 (*N*-deacetylase and *N*-sulfotransferase 1) is selectively reduced in GC B cells leading to attenuated IL-21 binding. Conversely, Ndst1 is upregulated in ASC, providing a further biochemical mechanism to balance the impact of IL-21 on GC recycling and differentiation. On the other hand, Duan and colleagues (84) have demonstrated that the B-cell response to IL-4 can be restricted by IL-4Rα expression on follicular dendritic cells (FDCs) that limits IL-4 availability within the GC environment. Thus, specific mechanisms operate to independently regulate both the IL-4 and IL-21 pathways within GC.

## Dysregulated IL-21 production in autoimmunity

Given the importance of IL-21 in shaping GC B-cell proliferation and fate, its tightly controlled expression and receptor sensitivity, and its intricate interplay with other pathways, dysregulation of IL-21 signaling is likely to have broad consequences. In this regard, elevated levels of IL-21 are known to be associated with autoimmunity both in humans and mice. In humans, overproduction of IL-21 has been noted in multiple autoimmune conditions including type 1 diabetes (85, 86), rheumatoid arthritis (87, 88) and systemic lupus erythematosus (SLE) (89–91). Indeed, recent work has identified that a variant in the regulatory region of a transcriptional repressor of IL-21, myocyte enhancer factor 2D (*Mef2d*), is associated with SLE (92, 93). In mice, IL-21 is overproduced in models of autoimmune diabetes (94, 95), and deficiency in IL-21R was found to prevent the development of diabetes in non-obese diabetic (NOD) mice, while transgenic expression of IL-21 in pancreatic islets was sufficient to trigger diabetes in non-autoimmune C57BL/6 mice (95, 96). IL-21 has also been shown to play a pathogenic role in mouse models of lupus (97, 98) and rheumatoid arthritis (99). We recently examined the role of IL-21 in mice experiencing systemic autoimmunity as a result of deficiency in the regulatory protein CTLA-4. These animals exhibit a lethal lymphoproliferative disease with autoimmune tissue infiltration in multiple organs and are characterized by spontaneous Tfh differentiation and GC formation with elevated IL-21 production (100). Consistent with the work discussed above, these chronic autoimmune GC harbored an augmented population of selected light zone GC B cells which was dependent on IL-21 signaling (54). Notably, an elegant study by Quast *et al*. (53) recently demonstrated that IL-21 had the capacity to regulate GC responses beyond cognate T-cell/B-cell interactions, increasing the potential for excess IL-21 to modulate the GC outcome in a bystander manner. Furthermore, while we have focused on the role of IL-21 within the GC in this review, it has emerged that appropriate regulation of IL-21 production in T cells collaborating with B cells at extrafollicular sites is also crucial for the prevention of autoimmunity (101, 102).

Collectively, these findings raise the possibility that the exacerbated IL-21 production that has been widely reported across many autoimmune conditions (85–91) may contribute to disease development by allowing selection, expansion and differentiation of GC B-cell clones that would otherwise be outcompeted (Fig. 2).

## Conclusions

The renewed interest in IL-21 biology has yielded significant advances in our understanding of IL-21-dependent regulation of humoral immunity. A series of highly complementary studies have now firmly established IL-21 as an essential regulator of GC dark zone formation. Moreover, reports of the profound GC dark zone loss in animals with defective IL-21 signaling have collectively helped reconcile the earlier observations of impaired GC expansion, maintenance, and reduced affinity maturation in IL-21−/− and IL-21R−/− mice. Importantly, IL-21 is now recognized as a key contributor to light zone GC B-cell positive selection for entry into the dark zone compartment as well as a principal driver of the dark zone GC B-cell inertial cycling. In addition to controlling the progression of the GC process, IL-21 signal strength has been pinpointed as a central threshold of GC-derived ASC differentiation. These latest insights into IL-21-dependent modulation of adaptive immunity provide fertile ground for future work aimed at untangling the contribution of exacerbated IL-21 production to the immune dysregulation observed in the context of autoimmunity. Furthermore, better understanding of the signals controlling B-cell fate decisions may aid the development of improved vaccination strategies.
